# Supplementary material for: Thermal effect on curved photovoltaic panels: Model validation and application in the Tabuk region
Source: PLoS One. 2022 Nov 2;17(11):e0275467. doi: 10.1371/journal.pone.0275467 (PMC9629591; doi:10.1371/journal.pone.0275467)
Supplement: Attachment — Submitted filename: Review Report-PLOS ONE.docx [file pone.0275467.s001.docx]

PLOS ONE

Thermal effect on curved photovoltaic panels: model validation and application in Tabuk region

Manuscript Number: PONE-D-22-16013

General: I, we, our, etc. should not be used.

Abstract: English editing is required.

To validate the method, ~~we have set up and studied~~ a 36-cell-50W solar panel with different radii of curvature is set up to assess solar power outputs under varying irradiance and temperature conditions.

For present application, the Tabuk region (Saudi Arabia) is chosen based on its location and climatic conditions.

**What are the climatic conditions made the basis for site selection and why not Dhahran or any other location?**

**Message is not meaningful:** Depending on the radius of curvature, an increase from 14.4 W to 25.8 W in the PV module power output was measured in the range of 307 W/m2 to 526 W/m2 of absorbed solar radiation.

Mixed tenses: In abstract, past and present tenses have been used. Please make uniform.

**Introduction: English editing is required.**

Many architects, designers, and manufacturers across the globe are investigating the usage of photovoltaics (PV) as a long-term energy source. ~~in their products.~~

The temperature of the solar module and the irradiance should be considered the most important—Did authors considered these two factors or it is a statement from literature?

In Section 2, first paragraph, the statements are conflicting or confusing. Are the authors explaining advantages of curves surfaces or disadvantages compared to other geometries?

The panels were oriented in an east-west direction and positioned at 28.38 as the latitude of Tabuk city. Shouldn’t it be south facing????

Is it geographic or Demographic position of Tabuk station???

How did the authors obtain Figure 4?

Please clarify the sentence: Figure 5 shows the interdependent variables 𝐼𝑜, 𝑎, and 𝑅𝑠 models which were simultaneously extracted versus temperature up to 65oC.

Figure 5: Authors show the modelled values and the curve fitting? Where is the measured data?

Same is the case in Figure 8.

Figure 9 and 10: These are the simulated values. Where are the measured values at different radius of curvatures?

Discussion section should be re-written for clarity and also English grammar should be rectified.

Following papers are suggested for enhancing the background of the topic of the manusvript:

1. Parametric Analysis and Optimization of a Novel Photovoltaic Trombe Wall System with Venetian Blinds: Experimental and Computational Study, **Case Studies in Thermal Engineering,** 34**,** 101958, **March 2022.**
2. A Comprehensive Global Review of Building Integrated Photovoltaic Systems, FME Transactions, March 2021, 49(2), 253-268.
3. Prospects of large-scale photovoltaic-based power plants in the Kingdom of Saudi Arabia, Engineering Reports, Article No. 12398, 1-16.
4. Global solar radiation and energy yield estimation from photovoltaic power plants for small load, **International Journal of Green Energy**, 14(4), 490-498. **(JIF = 1.171).** <http://dx.doi.org/10.1080/15435075.2016.1278374>
5. Performance evaluation of a PV (photovoltaic) module by back surface water-cooling for hot climatic conditions, **Energy – The International Journal 59, 445-453.**
6. Performance Evaluation of an off-grid Photovoltaic System in Saudi Arabia, **Energy – The International Journal**, v. 46(1), 451-458.
7. Usage of Photovoltaics in Automated Irrigation System, **Renewable Energy**, 23(1), (2001), pp. 17-26.
8. Feasibility study of the grid connected 10MW installed capacity PV power plants in Saudi Arabia. **Renewable and Sustainable Energy Reviews**, 80, 319 – 329.
9. Performance Evaluation of a PV Module Under Climatic Conditions of Dhahran, Saudi Arabia, **Energy Exploration & Exploitation** 33(6), 909-930.
