# Supplementary material for: Thermal effect on curved photovoltaic panels: Model validation and application in the Tabuk region
Source: PLoS One. 2022 Nov 2;17(11):e0275467. doi: 10.1371/journal.pone.0275467 (PMC9629591; doi:10.1371/journal.pone.0275467)
Supplement: Attachment — Submitted filename: Response to Reviewers.docx [file pone.0275467.s002.docx]

**Thermal effect on curved photovoltaic panels: model validation and application in the Tabuk region**

**PONE-D-22-16013**

**Response to Editor/Reviewers’ Comments:**

Dear Editor/Reviewers

The authors consider that all your comments and suggestions are of high scientific importance. Your ideas really helped us to substantially improve the quality of our manuscript. Thank You! We have corrected the errors that appeared in the manuscript and included the recommended changes in the revised manuscript (highlighted in **red** font). We hope the revised manuscript meets the expectations of the journal standards and will be considered for publication very soon.

Reviewer #1:

Comments to the Author

1) General: I, we, our, etc. should not be used.

**Response**: We appreciate your comment, and we have now corrected the manuscript accordingly.

2) Abstract: English editing is required.

**Response**: We appreciate your comment, and we have now edited the abstract accordingly.

3) To validate the method, ~~we have set up and studied~~ a 36-cell-50W solar panel with different radii of curvature is set up to assess solar power outputs under varying irradiance and temperature conditions.

For the present application, the Tabuk region (Saudi Arabia) is chosen based on its location and climatic conditions.

What are the climatic conditions made the basis for site selection and why not Dhahran or any other location?

**Response**: We appreciate your comment, and we have now corrected the text accordingly. As for the selection of Tabuk, it is considered a typical place in Saudi Arabia with a hot environment and where heat dissipation problems for PV panels are severe, the text is now changed.

4) **Message is not meaningful:** Depending on the radius of curvature, an increase from 14.4 W to 25.8 W in the PV module power output was measured in the range of 307 W/m2 to 526 W/m2 of absorbed solar radiation.

**Response**: We appreciate your comment, the text was confusing, and we have now made changes to this part of the results as follows:

The maximum power output of the PV module increases from 14.4 W to 25.8 W when the received solar power density varies from 307 W/m^2^ to 526 W/m^2^ depending on the level of curvature starting from a semi-cylindrical shape to a vaulted shape to a flat shape. Therefore, solar power reduction depends on the exposed (projected) area and not on the climatic condition – the amount of solar irradiance and ambient temperature. The measurements gave a percentage reduction in solar power ranging from 48.7% for semi-cylindrical shapes to 8.7% for stretched vaulted shapes. (This was demonstrated in the manuscript by conducting the experimental measurements under a solar irradiance of 1450 W/m^2^ and an ambient temperature of 35^o^C).

5) Mixed tenses: In abstract, past and present tenses have been used. Please make uniform.

**Response**: We appreciate your comment, and we have now edited the abstract accordingly.

6) Introduction: English editing is required.

**Response**: We appreciate your comment, and we have now edited the introduction accordingly.

7) Introduction: English editing is required.

Many architects, designers, and manufacturers across the globe are investigating the usage of photovoltaics (PV) as a long-term energy source. ~~in their products.~~

**Response**: We appreciate your comment, and we have now made the correction.

8) The temperature of the solar module and the irradiance should be considered the most important—Did authors considered these two factors or it is a statement from literature?

**Response**: We appreciate your comment, the authors considered the temperature and solar irradiance as the most important factors in the present investigation. We introduced for the first-time temperature-dependent parameters used in our modeling and simulation to support the researcher and power project developer.

9) In Section 2, first paragraph, the statements are conflicting or confusing. Are the authors explaining advantages of curves surfaces or disadvantages compared to other geometries?

**Response**: We appreciate your comment, the authors explained the advantages of the semi-cylindrical surface against the flat and fully cylindrical ones. We opted for a semi-cylindrical surface because it has a regular curved surface and possesses the characteristics of rounded and vaulted geometric shapes found in the KSA. The semi-cylindrical panel would occupy less footprint when compared to its planar counterpart having identical dimensions, same PV cell area, and working under the same conditions of irradiance and temperature. The sketch model in Figure 2 is now modified to illustrate the comparison between a semi-cylindrical panel and a planar counterpart. We then evaluated the percentage reduction in power density when using a curved surface as compared to a flat PV panel, under solar irradiance of 1450 W/m^2^, ambient temperature of 35^o^C, and 7% humidity (section 3).

10) The panels were oriented in an east-west direction and positioned at 28.38 as the latitude of Tabuk city. Shouldn’t it be south facing????

**Response**: We appreciate your comments, the text is now clarified to read:

The two identical PV panels were oriented in an east-west direction and positioned at 28.38 as the latitude of Tabuk city with their absorbing surfaces facing south and perpendicular to the sun's incoming rays.

11) Is it geographic or Demographic position of Tabuk station???

**Response**: We appreciate your comments, the text is now corrected to read geographic instead of demographic position.

12) How did the authors obtain Figure 4?

**Response**: We appreciate your comments, Figure 4 represents the experimental I-V curve under STC conditions, which is provided by the manufacturer of the MESM-50W PV module which was used in the investigation.

13) Please clarify the sentence: Figure 5 shows the interdependent variables 𝐼𝑜, 𝑎, and 𝑅𝑠 models which were simultaneously extracted versus temperature up to 65oC.

**Response**: We appreciate your comments, and we have made changes to the text accordingly.

The various interdependent parameters such as 𝐼_𝑝ℎ_, 𝑅_𝑠_, and 𝐼_𝑅_𝑠ℎwere determined following the same approach and methodology as recently reported by the authors in Ref 32. The novelty in this work stems from the introduction for the first-time of the PV panel temperature-dependent parameters in our modeling and simulation. This requires a simultaneous temperature dependent equations solving and solution of these parameters. Overall, 𝐼_𝑜_ 𝑎𝑛𝑑 𝑎, increases with temperature while 𝑅_𝑠_ decreases in the temperature range from 15^o^ C to 65 ^o^C. Below 15 ^o^C, we currently don’t have a realistic explanation as to why this anomaly is appearing at temperatures The obtained fitting models are just models which are well suited for this given experimental situation at lower thermal stress, but never perfectly. We believe that such parameters’ behavior might just result from the temperature dependence of another quantity, having a different physical origin than the true $a$ $andR_{s}$.

14) Figure 5: Authors show the modelled values and the curve fitting? Where is the measured data? Same is the case in Figure 8.

**Response**: We appreciate your comments, the answer to this is related to the previous comment.

The various interdependent parameters such as 𝐼_𝑝ℎ_, 𝑅_𝑠_, and 𝐼_𝑅_𝑠ℎwere determined following the same approach and methodology as recently reported by the authors in Ref 32. The approach is based on extracting all the needed parameters by exploiting the available parameters from the data sheets of commercial PV panels and by estimating the slopes at both short-circuit and open-circuit conditions of the current-voltage characteristic (Fig.4), usually provided by most solar panels manufacturers under standard test conditions (STC). Then, the established Simulink/MATLAB model based on the extracted experimental five parameters allows assessment of solar power outputs under any climatic conditions. The same thing applies to the maximum power point (MPP) values which is well known to increase linearly with the short circuit current, which in turn, increases linearly with irradiance.

15) Figure 9 and 10: These are the simulated values. Where are the measured values at different radius of curvatures?

**Response**: We appreciate your comments, we have validated our results with the product data from the manufacturer and which apply for both flat and curved identical PV panels. We have carried out measurements under a solar irradiance of 1450 W/m^2^, ambient temperature of 35^o^C, and 7% humidity (section 3) to evaluate the percentage reduction in power density when using a curved surface as compared to a flat PV panel with identical dimensions. The measurement gave a percentage reduction of 48.7% for the semi-cylindrical shape. Please note that this reduction depends on the geometry setting and not on the amount of solar irradiance under which we have conducted the experiment.

16) Discussion section should be re-written for clarity and also English grammar should be rectified.

**Response**: We appreciate your comments, we have improved the discussion section accordingly and polished the manuscript as well.

17) Following papers are suggested for enhancing the background of the topic of the manuscript:

**Response**: We appreciate your suggestion; we have included the referred papers to enhance our manuscript.
